# Patient Satisfaction with Anterior Bite Turbos: A Prospective Clinical Trial

**DOI:** 10.3390/dj13090412

**Published:** 2025-09-08

**Authors:** Fady Hussein Fahim, Donald Lloyd Baumann, Ahmed Othman, Reham M. Abdelsalam, Hamada Ahmed Deyab, Constantin von See, Dina Osman ElAbbasy

**Affiliations:** 1 Orthodontic Department, Faculty of Dentistry, Cairo University, Giza 12613, Egypt; fady.hussein@dentistry.cu.edu.eg (F.H.F.); dina.osman@dentistry.cu.edu.eg (D.O.E.); 2 Research Center for Digital Technologies in Dentistry and CAD/CAM, Department of Dentistry, Faculty of Medicine and Dentistry, Danube Private University, Steiner Landstraße 124, 3500 Krems an der Donau, Austria; ahmed.othman@dp-uni.ac.at (A.O.);; 3 Graduate Student, Department of Orthodontic and Orofacial Orthopedics, Boston University, Boston, MA 02215, USA; rsalam@bu.edu; 4 Orthodontic Department, Borg Al Arab Technological University, International Dental Ambassador for RCSEd, Examiner Royal College of Physicians and Surgeons of Glasgow, Glasgow G2 5RJ, UK

**Keywords:** mesh terms, over bite, deep bite, overjet, dental overjet, satisfaction, patient, assessments, patient outcome, outcome assessments, patient, patient outcome assessments, assessment, patient outcomes, outcomes assessment, patient-centered outcomes research, patient centered outcomes research, research, patient-centered outcomes

## Abstract

**Background**: Bonded composite bite turbos are an efficient tool in the treatment of deep bite malocclusion. Patient satisfaction with orthodontic appliances has been correlated with treatment success. The aim of this study is to evaluate the level of patient satisfaction associated with bonded composite anterior bite turbos in deep bite treatment. **Materials and Methods**: This study was a one-arm prospective clinical study. Sixty patients, younger than 25 years old with permanent dentition and increased anterior overbite were treated with composite bite turbos bonded to the maxillary central incisors. A questionnaire form using the Likert scale with five responses (Very Unpleasant, Unpleasant, Acceptable, Pleasant, and Very Pleasant) was used by patients to document their satisfaction and feedback regarding the bite turbos after 1 week and 1 month. Statistical analyses with the chi-squared test was used to analyze the data for statistical significance. **Results**: Spearman’s correlation coefficient was used to determine the correlation between age and satisfaction score, while the Mann–Whitney U test was used to compare the satisfaction scores between males and females. There was a statistically significant difference between all patient responses after 1 week and 1 month. A significant decline occurred in the unpleasant response from 10% after 1 week to 0% after 1 month; acceptable response from 36.7% after 1 week to 3.3% after 1 month. There was a statistically significant increase in the pleasant and very pleasant responses. Minor speech and eating difficulties were reported during the first week by 46.7% of patients. **Conclusions**: Anterior bite turbos made from Triad Gel and bonded to palatal surfaces of both maxillary central incisors using a Mini-Mold tip with 5 mm depth tip will most likely lead to an acceptance rate of 90% after 1 week and 100% acceptance rate after 1 month, without any problems in 53.3% of the patients. Short follow-up periods and lack of control group are the main limitations in this study. Trial registration: Registered at ClinicalTrials.gov (NCT07143916).

## 1. Introduction

Patients often seek orthodontic treatment to improve their esthetic appearance. The ideal clinical management of patients undergoing fixed orthodontic therapy greatly depends on the skills of the orthodontist. However, factors contributing to the success of orthodontic treatment include patient motivation, cooperation, and compliance. These factors also rely on the level of patient satisfaction with regards to discomfort or pain during treatment [1,2,3].

Along the course of orthodontic treatment, nearly all patients have declared to have experienced some degree of discomfort or pain [4,5]. Discomfort is defined as light tactile pressure on the teeth or oral mucosa, stretching of soft tissues, teeth sensitivity, and pain [1,6,7]. Patients undergoing orthodontic treatment encounter eating difficulties and pain, which declines as time passes [7]. Although the pain is mild and concise, it may drive many patients to discontinue treatment [1,8,9]. Discomfort may lead to eating limitations to a soft diet which can result in poor nutrition in orthodontic patients, and consequently affects the biological response of the bone and the periodontal ligament to orthodontic forces [10].

A deep curve of spee is usually associated with an increased anterior overbite. [11]. The correction of deep bite is one of the objectives of orthodontic treatment. Treatment strategies depend on the etiology, and include extrusion of posterior segment, intrusion and/or proclination of anterior teeth, or a combination of both [12,13,14,15,16,17]. A range of appliances can be used for the treatment of dental deep bite, such as reverse curve of spee archwires [11,14,16,18], cervical pull headgear [19] and anterior removable or fixed acrylic bite plates [14,20].

Anterior bite plates, whether fixed or removable, work by disarticulation of the posterior teeth resulting in their passive eruption and extrusion. They are beneficial in patients with reduced lower anterior facial height. However, they are not without disadvantages. Removable anterior bite plates require patient compliance and cooperation, as well as periodic adjustments to allow for orthodontic tooth movements. On the contrary, fixed anterior bite plates are a good alternative but can cause soft-tissue irritation and limitation of tooth movements [20,21].

To overcome the shortcomings of fixed and removable anterior bite plates, Joe Mayes of Ormco [22] introduced bonded bite turbos in 1997 as an alternative by bonding them to the palatal surface of maxillary incisor teeth. They were named “Bite Turbos” as they immediately open the bite. They were also named anterior bite elevators and became popular in use over the last 10 years [23]. They are made of either metal or composite resin [20,23]. The advantages of bite turbos include smaller size, better hygiene, simplicity, and durability. Their removal is also as simple as removal and cleanup of a lingual bracket [20,23]. However, due to variations in the palatal anatomy of maxillary incisors, their placement can frequently be a bit tricky. Other common drawbacks reported by patients include lisping, pain, discomfort, difficulty in swallowing, and intense tooth vibrations, as reported by Kravitz et al. [20]. Significant patient compliance is required for orthodontic treatment, and events like this can have a negative impact on patient acceptance [23].

Along with evaluating treatment efficiency, it is also necessary to assess to what extent patients can tolerate the intended treatment processes. Scales for assessment, such as Likert [24] and the VAS (Visual Analogue Scale) [25], are beneficial tools for evaluating patient discomfort or pain during treatment [1,24,26,27,28].

Since bite planes are a crucial tool for deep bite correction, and many orthodontists prefer the bonded bite turbos because they are easy to make with no extra laboratory work, it is mandatory to investigate the level of acceptance of orthodontic patients. To our knowledge, no previous studies have investigated patient satisfaction, tolerance, and feedback regarding bite turbos. Therefore, the aim of this study was to investigate the degree of patient satisfaction and to evaluate the patient feedback regarding the clinical use of bonded composite anterior bite turbos.

## 2. Materials and Methods

### 2.1. Study Design

This study was a one-arm prospective clinical study. It was conducted in accordance with the Declaration of Helsinki (2013 revision). The protocol was approved by the Research Ethics Committee of Cairo University number 26-6-2023, and registered on ClinicalTrials.gov (NCT07143916). Recruitment was performed and the study started on first of January 2024.

### 2.2. Sample Size Calculation

According to previous results [29] for the same endpoint, the satisfaction level for the bite turbo was 85.8%, assuming 80% of patients treated with the bite turbo had no effect on the satisfaction levels of patients compared to 20% with a low satisfaction level. Therefore to achieve a power of 80% for the study at a level of significance = 0.05, the required total sample size was 54 patients (adding 10% drop out) to obtain 60 patients. Therefore, a total of 60 patients were selected from the outpatient clinic of the Department of Orthodontics, Cairo University.

### 2.3. Inclusion and Exclusion Criteria

The selection criteria were as follows:

Inclusion criteria:

1. Patients seeking orthodontic treatment;
2. Good oral hygiene;
3. Decreased lower anterior facial height;
4. Increased anterior overbite;
5. Full permanent dentition;
6. Overjet less than 5 mm.

Exclusion criteria:

1. Patients older than 25 years;
2. Deciduous and mixed dentition;
3. Patients with periodontal disease.

### 2.4. Demographic and Clinical Criteria of Participants

Sixty patients with increased overbite and decreased vertical dimension were selected. The sample consisted of 18 males and 42 females with a mean age of 19 (±3.74) years (Table 1).

### 2.5. Informed Consent

A consent form was signed by all patients after explaining the purpose of bite turbos and the whole bonding procedure. Before initiating treatment, full orthodontic records were taken for each patient including panoramic and lateral cephalometric radiographs, and study models, as well as intraoral and extraoral photographs.

The clinical steps were as follows:

1. The maxillary arch was bonded, and 0.014 or 0.016 NiTi archwires were ligated.
2. After one month, the bite turbos were bonded on the palatal surfaces of the maxillary incisors
3. The lower arch was delayed until the deep overbite was corrected, mostly by molar extrusion.

The first author of this study, (FF), delivered the intervention for all the samples at the Orthodontic Department of Cairo University. A one-month interval was intended between bonding of the maxillary arch and bonding of the anterior bite turbos to avoid overlap between patient discomfort accompanied after the initial bonding visit and the acceptance level of the bite turbos. For the same reason, bonding of the lower arch was delayed until the satisfaction survey was completed and delivered to ensure absence of any overlap of the usual patient discomfort after the bonding visit and the bite turbos level of satisfaction. It is worth mentioning that the maxillary archwire was not changed in the visit for bonding the anterior bite turbos for the same reason stated above.

**Figure 1:**
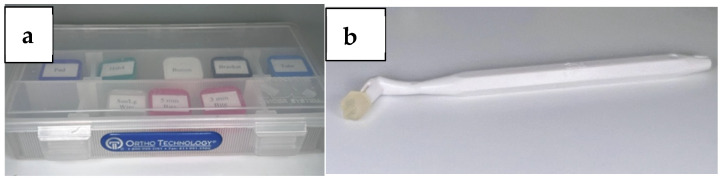
(**a**) Mini-Mold starter kit; (**b**) Mini-Mold tip handle with 5 mm bite ramp tip.

**Figure 2:**
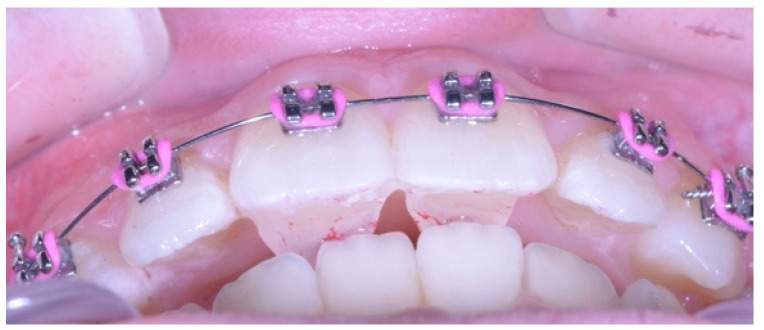
Anterior bite turbos bonded on palatal surfaces of maxillary incisors using Triad Gel light cure.

Triad Gel™ light cure (Dentsply Sirona, York, PA, USA) was used to bond the anterior bite turbos. A Mini-Mold tip (Ortho Technology, 1822 Aston Avenue, Carlsbad, CA, USA) with a 5 mm depth tip was used to standardize the depth of the bonded anterior bite turbos (Figure 1 and Figure 2).

After polishing and acid etching the palatal fossae of the maxillary central incisors, A single bond universal adhesive (3M ESPE, Seefeld, Germany) was added, followed by packing the light cure Triad Gel inside the molds in such a way to avoid air bubbles. The mold was applied on the palatal surface at the palatal fossa of the maxillary central incisor just above the cingulum using light finger pressure to ensure contact of the mold and band cement on the enamel, and then light cured by the dental assistant using the light cure unit (3M™ Elipar™ DeepCure -L LED Curing Light, Germany) for 10 s through the transparent mold. Afterwards, the mold was removed and the bite turbo was additionally cured for another 10 s. Finally, the patient was asked to bite to ensure that the turbo depth was sufficient for proper contact with the lower central incisors (Figure 2). It is worth mentioning that both bite turbos were bonded in the same single visit. The bonded bite turbos were intended to last not only for one month, which is period of the study, but during the entire orthodontic treatment until the deep overbite was corrected.

**Figure 3:**
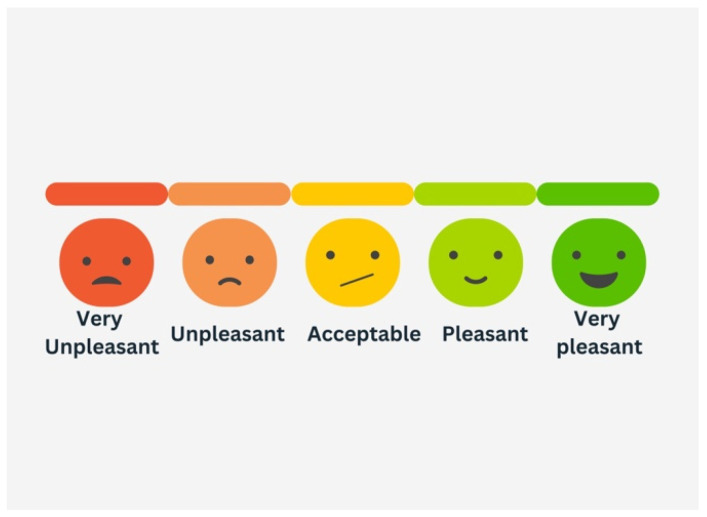
Satisfaction survey forms based on Likert scale to state their acceptance level according to these 5 responses: Very Unpleasant, Unpleasant, Acceptable, Pleasant, and Very Pleasant.

Immediately after the bonding visit, all patients received two printed satisfaction survey forms based on the Likert scale [24] to state their acceptance level according to these 5 responses: Very Unpleasant, Unpleasant, Acceptable, Pleasant, and Very Pleasant (Figure 3). The patients were asked to document their acceptance level after 1 week and after 1 month. Additionally, patients who were dissatisfied with the bite turbos were asked to write down in the printed survey form any specific problems encountered, such as speech or eating difficulties. Clear instructions were given with the satisfaction survey form that it is mandatory to assess your acceptance level for the turbos only and for nothing else.

## 3. Statistical Analysis

Data were coded and entered using the statistical package SPSS version 22; IBM Corporation, Armonk, NY, USA. Categorical data were expressed as frequency and analyzed by the chi-squares test. A *p*-value less than or equal to 0.05 was considered statistically significant. Spearman’s correlation coefficient was used to determine the correlation between age and satisfaction score. The Mann–Whitney U test was used to compare the satisfaction scores between males and females.

## 4. Results

**Figure 4:**
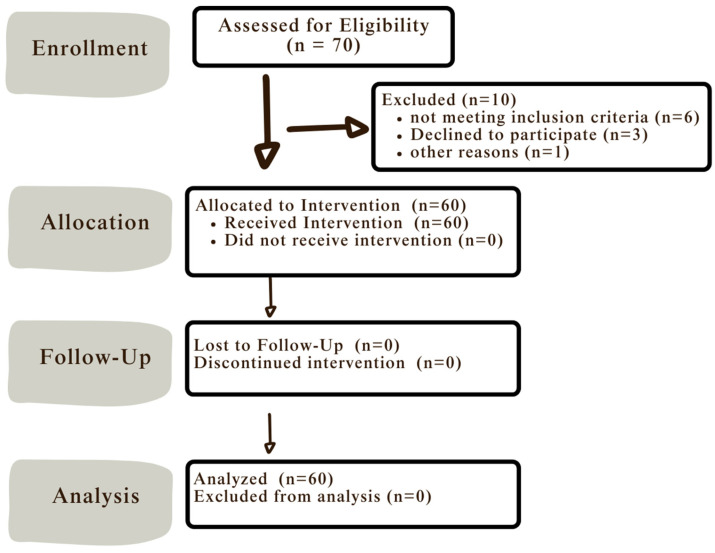
Patient flow chart. n: the number of patients enrolled.

Patient Flow: Initially, 70 patients were assessed for eligibility; of these, 10 were excluded. Consequently, a total of 60 patients were enrolled in one group (n = 60). All patients received their allocated intervention (bonded bite turbos on the palatal surfaces of the maxillary central incisors). All patients completed the one week and one month follow-up period, and data from all 60 patients were included in the final analysis, as summarized in Figure 4.

This study was conducted on 60 patients, 18 males and 42 females with an age range 13 to 25 years and a mean age of 19 ± 3.74 (Table 1). The patients’ general experience and feedback were assessed at 1 week and at 1 month following bonding of the anterior resin bite turbos (Table 2). The Likert scale was categorized to five responses: Very unpleasant, Unpleasant, Acceptable, Pleasant, and Very Pleasant.

It is noteworthy that the acceptance rate reached 90% after 1 week and increased to 100% after one month, with 53.3% of patients reporting no issues. Additionally, it is a remarkable finding that not one of the patients reported a very unpleasant experience at either the one-week or one-month evaluation (0.0%).

At the 1 week evaluation, 6 patients reported an unpleasant experience (10.0%), while 22 patients reported an acceptable experience (36.7%). A total of 18 patients (30.0%) reported a pleasant experience, and 14 patients (23.3%) reported a very pleasant experience after the first week.

After 1 month, the evaluation revealed that no patients reported an unpleasant experience (0.0%), whereas only two patients reported an acceptable experience (3.3%). A total of 27 patients (45.0%) reported a pleasant experience, and 31 patients (51.7%) reported a very pleasant experience after 1 month of treatment with the bite turbos. There was a statistically significant difference between the results for each category after 1 week and after 1 month with a *p*-value <0.001.

Regarding the patients’ specific complaints during the first week (Table 3), 16 patients reported eating difficulties (26.7%), while 12 patients reported speech problems (20%). A total of 32 patients (53.3%) reported no complaints with regards to eating or speech. There was a statistically significant difference between the results for each category with a *p*-value = 0.004. However, no specific complaints were reported by the patients after 1 month.

After one week as well as after one month, there was a statistically significant inverse (negative) correlation between age and satisfaction score. Older age was associated with lower satisfaction scores and vice versa (Table 4). In addition, after one week as well as after one month, there was no statistically significant difference between the satisfaction scores of males and females (Table 5).

## 5. Discussion

This prospective clinical study assessed the level of satisfaction of patients with deep bite malocclusion regarding anterior bite turbos bonded to their maxillary central incisors. To the best of our knowledge, no study has addressed this subject in the literature. Furthermore, no previous study has evaluated patient satisfaction with anterior removable bite plates. As previously reported, the degree of patient satisfaction with certain orthodontic appliances is crucial in determining the success of the treatment plan [30,31]. Hence, our study aimed to investigate this criterion associated with orthodontic treatment.

This study was performed on an Egyptian population, males and females, with a mean age of 19 years. Bonded bite turbos are frequently used nowadays to treat increased overbites with decreased vertical dimension. The length of the follow up period in this study was only 1 month. Therefore, we believe that this study can be generalized in other populations.

Bonding anterior bite turbos on the palatal surface of maxillary central incisors can be useful as anterior removable bite plates in causing posterior teeth disarticulation necessary for deep bite correction. Space will be created posteriorly into which posterior segments extrude until they reach the new occlusal plane [32]. Previous research has shown that they produce small relative intrusion of the maxillary and mandibular incisors and minor lower incisor proclination. They are advantageous in that they avoid compromised lower bracket positioning as well as gingival and periodontal problems which occur when brackets are bonded too far gingivally [33]. Additionally, they assist in the bonding of the lower second molars early in treatment which provides a lever arm for lower posterior teeth extrusion [20]. Bite turbos are ideally bonded to the palatal surfaces of both maxillary central incisors for better distribution of occlusal forces. Their occlusal table is horizontal so no distal force is placed on the mandible [20].

All the patients in this study had anterior deep bite malocclusion with normal/short anterior facial height. Therefore, we bonded the bite turbos anteriorly on the palatal surface of the maxillary central incisors to accommodate any increase in facial height due to the extrusion of posterior teeth [23]. Patients with compromised periodontium were excluded from this study to avoid further periodontal breakdown from increased forces on the lower central incisors due to contact with the bite turbos.

Bite turbos are available in different lengths depending on the amount of overjet. In patients with a normal overjet, a length of 3 mm is used while a larger overjet entails the use of a length of 5 mm to contact the lower incisors [23,32]. Since the average amount of anterior overjet of the patients in this study was less than 5 mm, we preferred to use the long 5 mm mold over the short 3 mm mold.

Bite turbos can also be fabricated from metal or resin material, such as acrylic gels (Triad Gel) or band cements (Ultra-Band-Lok), that have low quartz content, thereby minimizing the risk of abrasion on opposing teeth [20,22]. Therefore, in our study, we preferred to use Triad Gel (Dentsply Sirona) to bond the anterior bite turbos because of the low amount of quartz particles that would not wear the opposing enamel. According to our personal clinical experience, composite bite turbos were chosen over the metal ones due to the high failure rate of metal turbos and the uncomfortable feel that the patients experience from contact of the lower central incisors with metal.

As per previous studies [20,22], composite turbos can be directly applied on the tooth surface utilizing a syringe or using a bond brush. Despite the simplicity of this technique, it has a significant drawback, which is the lack of an appropriate measurement of the amount applied as well as the anatomical variations in the palatal surfaces of the maxillary teeth [34]. This creates uneven amounts of resin on the teeth thus exposing them to various levels of occlusal forces. Hence, in this study prefabricated mini-mold bite ramps were used which maintained accurate bonding of identical and well-formed bite turbos. This allowed for uniform occlusal force levels upon contact with the lower central incisors [32].

The amount of disocclusion produced for the posterior teeth was 3 mm on average, which was reduced to 1 mm on average after 1 month. It is worth mentioning that no bonding failures of the bite turbos was reported neither after 1 week, nor after 1 month.

The Likert scale is the most commonly used scaling and rating technique [25,35] in health research studies [35,36]. It has also been widely used in the evaluation of dentofacial esthetics. Another commonly used scale is the VAS, which is implemented in studies that assess subjective experiences like pain [24]. It is composed of a 10 cm graded line on which the extreme points at each end are either descriptive words of opposite statements or the minimum and maximum extremes of the aspect being measured, such as “no pain” or “severe pain” [25].

Most evaluators, including laypeople, oral surgeons, and orthodontists favor the five-point Likert scale over VAS for assessing facial pleasantness following orthodontics, as it is considered easier to use and more effective in conveying their opinions [24]. Regarding AI applications in orthodontics, a five-point Likert scale has been employed to evaluate accuracy, reliability, quality, and readability of responses generated by ChatGPT-3.5, ChatGPT-4, Gemini, and Copilot in relation to orthodontic-related questions [37]. According to a systematic review by Marzuki et al. [36], Likert was preferred by both clinicians and laypeople for its simplicity and ability to capture opinions more effectively. This was also corroborated by Dourado et al. [24], who noted that VAS required more effort to understand, especially for respondents with limited familiarity with the scale. Furthermore, a systematic review on the impact of functional appliances on the soft tissues included studies that used attractiveness assessments based on either the VAS or Likert scale as eligible for inclusion [26]. Therefore, the Likert scale was selected as the preferred tool in the present study, with responses categorized into five levels.

According to the results illustrated in Table 2, a significant improvement in the degree of patient acceptance and satisfaction with the bite turbos was observed. There was a statistically significant reduction in the number of patients who considered their experience with the bite turbos as unpleasant, decreasing from six patients after 1 week to zero patients after 1 month. Additionally, there was a statistically significant surge in the number of patients whose experience was very pleasant, increasing from 14 patients after one week to 31 patients after 1 month. Similarly, a significant increase occurred in the number of patients who had a pleasant experience with the bite turbos, rising from 18 patients after 1 week to 27 patients after 1 month. There was a reduction in the number of patients who reported an acceptable experience with the bite turbos, decreasing from 22 patients after 1 week to only 2 patients after 1 month. Only 26.7% of the sample reported eating problems, and 20% reported speech problems, that faded away gradually with time.

As shown in Table 4, older patients were associated with lower satisfaction scores, whereas younger patients demonstrated higher acceptance rates, a finding that aligns with expectations. Additionally, no significant difference was observed between males and females, as both groups reported comparable satisfaction scores.

## 6. Limitations of the Study

This study was performed to assess the satisfaction level of only two bonded bite turbos on the palatal surfaces of maxillary central incisors only. Increasing the number of bonded bite turbos on more teeth or changing the location on other teeth might result in different findings. In addition, metal bite turbos have a different feeling than the Triad Gel light cure; therefore different observations will be expected.

Since there was a high level of acceptance after one month, the results are expected to be the same with longer follow ups. However, this assumption needs long term investigation. 

There was no control group in this study. We believe that there will be no added value because we measured level of patient acceptance rather than treatment effects.

Therefore, a short follow-up period and the lack of a control group are the main limitations in this study.

## 7. Recommendations for Future Research

The location of the anterior bonded turbos, either on central incisors, lateral incisors, or canines may have a clinical impact and might also affect the level of patient acceptance; therefore, it is worthy of more research.

The material of anterior bonded turbos, either metal, compomers, resin-modified glass ionomer, or composite, might influence the “patient feel” while biting on them; therefore, it is worthy of further investigations.

Some clinicians prefer to bond anterior bonded turbos with greater length (depth), which may affect fracture rate and patience acceptance; therefore, future research is recommended.

Long term studies (6 months, 1 year, 2 years) and randomized controlled trials will definitely be an added value in the orthodontic literature.

## 8. Conclusions

Anterior bite turbos made from Triad Gel and bonded to palatal surfaces of both maxillary central incisors using Mini-Mold with a 5-mm depth tip will most likely lead to an acceptance rate of 90% after 1 week and 100% acceptance rate after 1 month, without any problems in 53.3% of the patients.

## Figures and Tables

**Table 1:** Descriptive data of the sample.

**Number of Patients** **(N) = 60**	**Sex**		**Age**
	**Male**	**Female**	**19 (±3.74)**
	18 (30%)	42 (70%)	

**Table 2:** Patient satisfaction level regarding the bite turbos according to the Likert scale after 1 week and after 1 month.

			After 1 Week	After 1 Month	*p*-Value
**Likert scale**	**Very Unpleasant**	**N**	0	0	
		**%**	0%	0%	
	**Unpleasant**	**N**	6	0	<0.001
		**%**	10.0%	0.0%	
	**Acceptable**	**N**	22	2	
		**%**	36.7%	3.3%	
	**Pleasant**	**N**	18	27	
		**%**	30.0%	45.0%	
	**Very pleasant**	**N**	14	31	
		**%**	23.3%	51.7%	

N = Number.

**Table 3:** Specific patient complaints regarding bite turbos.

Specific Patients’ Complaints	N	%	*p*-Value
Can’t eat	16	26.7	0.004
Speech	12	20	
Nothing reported	32	53.3	

**Table 4:** Spearman’s correlation coefficient for the correlation between age and satisfaction scores.

Time	Correlation Coefficient (ρ)	*p*-Value
1 week	−0.257	0.047 *
1 month	−0.299	0.020 *

*: Significant at *p* ≤ 0.05.

**Table 5:** Descriptive statistics and results of the Mann–Whitney U test for comparison between satisfaction scores of males and females.

Time	Males (n = 18)		Females (n = 42)		*p*-Value
	Median (Range)	Mean (SD)	Median (Range)	Mean (SD)	
1 week	4 (2, 5)	3.9 (1)	3.5 (2, 5)	3.6 (0.9)	0.250
1 month	5 (3, 5)	4.6 (0.6)	4 (3, 5)	4.4 (0.5)	0.183

## Data Availability

The raw data supporting the conclusions of this article will be made available by the authors upon request.
